# Long-term pasireotide therapy in acromegaly: extensive real-life experience of a referral center

**DOI:** 10.1007/s40618-023-02299-7

**Published:** 2024-03-26

**Authors:** R. Pirchio, R. S. Auriemma, A. Vergura, R. Pivonello, A. Colao

**Affiliations:** 1 Dipartimento di Medicina Clinica e Chirurgia, Sezione di Endocrinologia, Diabetologia, Andrologia e Nutrizione, Università Federico II di Napoli, Via S. Pansini 5, 80131 Naples, Italy; 2 UNESCO Chair for Health Education and Sustainable Development, Federico II University, Naples, Italy

**Keywords:** Acromegaly, Pasireotide, Pituitary tumor, Glucose, Insulin, Lipid

## Abstract

**Purpose:**

Pasireotide is a novel therapeutic option for patients with acromegaly resistant to first-generation somatostatin receptor ligands. To date, real-life data are still scant, therefore, the aim of the current study is to evaluate the impact of long-term pasireotide therapy on disease control, pituitary tumor size, gluco-insulinemic and lipid profile in a real-life setting.

**Methods:**

Retrospective study of data prospectively collected, evaluating hormonal, tumoral, and metabolic data of 28 patients with acromegaly administered with pasireotide in a pituitary tertiary referral center.

**Results:**

Within the first 12 months of treatment, 70.4% of patients achieved normal IGF-I levels, which was maintained at 36-month evaluation in these responders patients. Patients who started with pasireotide 60 mg monthly exhibited significantly lower IGF-I levels after 36 months (*p* = 0.05) as compared to patients administered first with pasireotide 20 or 40 mg monthly. The maximal tumoral diameter was significantly decreased after 12 months of pasireotide (*p* < 0.001) and a further reduction was registered throughout the following months, with 41.2% of patients achieving a significant reduction (> 25% of baseline measurement) after 36 months of treatment. Fasting glucose significantly increased during the first 6 months (*p* < 0.001) with a gradual rise in diabetes prevalence during the following months, resulting diabetes prevalence after 36 months of pasireotide significantly increased compared to baseline (*p* = 0.003), although with glycated hemoglobin levels within the normal range. Diabetes was managed using oral glucose-lowering drugs or glucagon-like peptide 1 agonists, with no patient requiring insulin therapy. Pasireotide improved lipid profile, mainly during the first 12 months of treatment, by increasing HDL and decreasing triglycerides levels.

**Conclusion:**

Pasireotide is effective and safe in the long-term. Hyperglycemia is a common event and is manageable even without insulin treatment.

## Introduction

Acromegaly is a rare endocrine disease caused by a GH-secreting pituitary adenoma in most cases [1]. Its therapeutic approach is multidimensional and tailored to the patient [1]. Pasireotide (PAS), second-generation somatostatin receptor ligand (SRL), is a novel valid therapeutic option for patients with acromegaly resistant to first-generation SRLs [2]. PAS has been proven to exhibit a different somatostatin receptor (SSTR) binding profile as compared to first-generation SRLs, which allows overcoming the resistance due to predominant SSTR5 expression, which represents the main reason for resistance to first-generation SRLs. Indeed, PAS has approximately 30-, 11-, and 158-fold higher functional activity than octreotide LAR on SSTR1, SSTR3, and SSTR5, respectively, and seven-fold lower activity on SSTR2 [3, 4]. Two independent phase III clinical trials have demonstrated PAS to be more effective in normalizing both GH and IGF-I levels in either SRLs-naïve patients (*C2305*) [5] and in those resistant to first-generation SRLs (*C2402 PAOLA study*) [6]. Furthermore, a significant decrease in pituitary tumor volume (i.e., > 25%) occurred in a higher proportion of patients receiving PAS compared to those receiving first-generation SRLs [5–7], therefore, PAS has been supposed to exert further anti-proliferative mechanisms than first-generation SRLs [8]. These promising results have been subsequently confirmed also in real-life setting [9]. Long-term disease control has been reported in 30–50% of patients in the extension of the PAOLA study up to 67 months [10] and in 79% up to 50 months in a multicenter study [11].

However, biochemical and tumoral effectiveness of PAS in acromegaly is burdened by the negative impact on glucose tolerance. Indeed, PAS-induced hyperglycemia is the most common adverse event, occurring in 57% and 64% of patients in *C2305* [5] and *C2402* [6] studies, respectively. Similar findings were reported in real-life setting, where the prevalence of glucose impairment has been reported to increase from 61.3% at baseline to 92.4% after 6 months of PAS [9]. Notably, PAS has been suggested to cause secondary diabetes, which is reversible after discontinuation of PAS. Differently, no data about PAS influence on lipid metabolism in acromegaly are nowadays available.

To the authors’ knowledge, real-life data about long-term efficacy and safety of PAS in acromegaly are very limited [9, 11] and no data are currently available regarding the effects on the lipid profile of PAS treatment in patients with acromegaly. Therefore, the present retrospective study of data prospectively collected aimed at investigating the impact of long-term PAS therapy (≥ 36 months, up to 90 months) on disease control, pituitary tumor size, gluco-insulinemic and lipid profile in PAS naïve acromegaly patients in a real-life setting.

## Patients and methods

### Inclusion and exclusion criteria

The current retrospective study of data prospectively collected included adult patients with a diagnosis of acromegaly according to international guidelines [12]. Inclusion criteria were: (1) patients resistant to long-term high-dose treatment with first-generation SRLs in monotherapy or combined with cabergoline (CAB) or pegvisomant (PEG) in those with partial resistance to SLRs or intolerant to long-term high-dose treatment with first-generation SRLs monotherapy; (2) stable size of the pituitary tumor for at least 12 months before study entry, as documented by magnetic resonance imaging [MRI]; and (3) patients on stable hormone replacement therapy for hypopituitarism for at least 6 months before study entry. Exclusion criteria were: (1) chronic hepatitis or liver failure; (2) suspicion of drug or alcohol abuse; (3) any other condition resulting in abnormal GH and IGF-I levels, such as severe renal disease as well as malnutrition; (4) radiotherapy performed during or one year before treatment with PAS; and (5) incomplete data during follow-up.

### Patients

Twenty-eight consecutive patients (10 males, 18 females, age 47.8 ± 12.1 years) were administered with PAS at the Section of Endocrinology at University “Federico II” of Naples between January 2015 and May 2023.

**Fig. 1:**
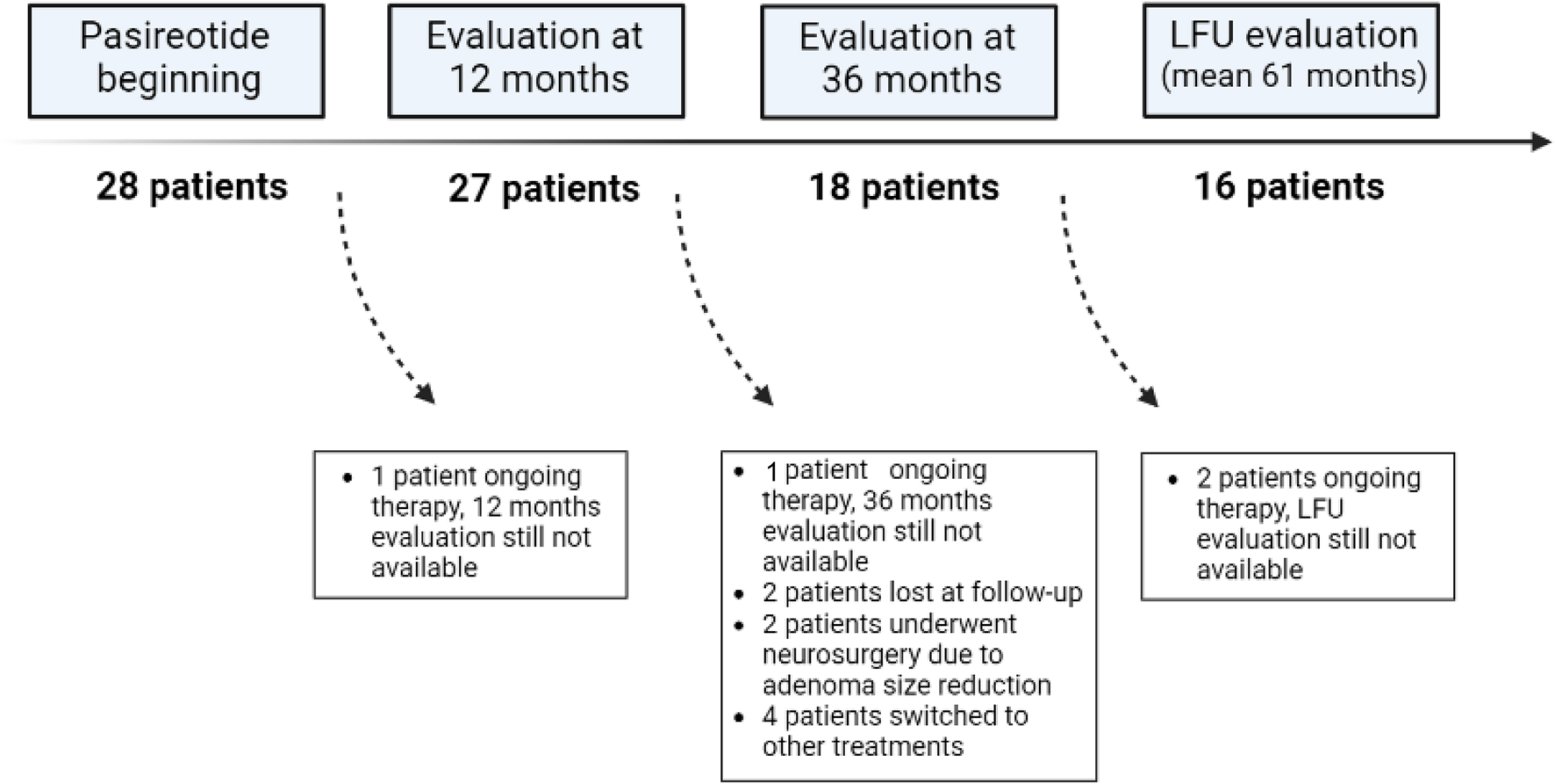
Details of the study population

Among these patients, 18 (64.3%) received PAS treatment for at least 36 months and 16 (57.1%) were treated with PAS for a longer period (range 42–90 months, Fig. 1). Patients’ profile at the study entry is detailed in Table 1.

**Table 1:** Patients’ profile at study entry

Patients, *n*	28
Age, years	47.8 ± 12.1
Male/female, *n*	10/18
Age at diagnosis, years	40.5 ± 11.6
Microadenoma at diagnosis, *n* (%)	2 (7.1)
Macroadenoma at diagnosis, *n* (%)	26 (92.9)
Prior to pasireotide starting	
GH, ng/ml	4.1 ± 3.3
IGF-I, ng/ml	458.4 ± 184.8
IGF-I, x ULN	1.8 ± 0.6
Maximal tumor diameter, mm	17 ± 8.7
Empty sella, *n* (%)	1 (3.6)
Disease duration, months	182.9 ± 82.7
Previous therapies	
Neurosurgery, *n* (%)	20 (74.1)
Second neurosurgery, *n* (%)	1 (3.6)
Third neurosurgery, *n* (%)	1 (3.6)
Radiotherapy, *n* (%)	2 (7.1)
SRLs monotherapy, *n* (%)	21 (75)
SRLs + cabergoline, *n* (%)	2 (7.1)
SRLs + PEG, *n* (%)	5 (17.9)

### Study protocol

The current retrospective study aimed at investigating the impact of PAS therapy on disease control, pituitary tumor size, gluco-insulinemic, and lipid profile in PAS naïve acromegaly patients in a real-life setting, particularly focusing on the long-term therapy defined as a treatment duration of at least 36 months.

As per internal protocol, at diagnosis and thereafter at 3–6 months intervals, all patients were admitted to the hospital for a complete physical, biochemical, and endocrine examination. Four time-points have been considered for the present study: prior to PAS starting, namely baseline, and evaluations at 6-, 12-, and 36-month of PAS treatment; last follow-up (LFU) evaluation has also been included for the patients with longer treatment duration. At each time point, GH and IGF-I levels were assessed in all patients, with the exception of GH at baseline in patients administered with PEG before starting PAS to avoid any possible interference of PEG molecule in the assessment of this hormone [13]. In the current study, disease control has been defined as normal IGF-I adjusted for age and sex (i.e., ≤ 1 × ULN) according to the last international guidelines [12]. The prevalence of patients achieving also a random GH < 2.5 ng/ml, according to *C2305* and *C2402* studies, or random GH < 1 ng/ml, as the more recent guidelines [12], has been also provided in Table 2. Biochemical parameters, including fasting glucose (FG) and insulin (FI), glycated hemoglobin (HbA_1c_), total- cholesterol, LDL-cholesterol (LDL), HDL-cholesterol (HDL), and triglycerides, were assessed at each visit. Pituitary MRI with gadolinium was assessed before starting PAS treatment and subsequently every year to evaluate pituitary tumor size and its changes during this treatment. For the current study, the maximal tumor diameter (MTD) has been considered for all patients.

**Table 2:** Biochemical and hormonal parameters and maximal tumor volume (MTD) prior to pasireotide and after 6, 12, and 36 months of treatment and at last follow-up (LFU)

	Prior to pasireotide (A) *n* = 28	After 6 months (B) *n* = 28	After 12 months (C) *n* = 27	After 36 months (D) *n* = 18	LFU (E) n = 16	p (A vs B)	p (B vs C)	p (A vs C)	p (A vs D)	p (C vs D)	p (D vs E)
GH, ng/ml	4.1 ± 3.3*	4.5 ± 1.4	2.4 ± 3.7	1.6 ± 0.9	1.5 ± 0.9	** < 0.001**	0.25	** < 0.001**	** < 0.001**	0.10	0.86
IGF-I, x ULN	1.8 ± 0.6	1 ± 0.4	1 ± 0.6	0.7 ± 0.2	0.6 ± 0.2	**0.004**	0.903	** < 0.001**	**0.03**	0.569	0.790
IGF-I < 1 ULN, *n* (%)	2 (7.1)	16 (57,1)	19 (70.4)	18 (100)	15 (93.7)	**0.004**	**–**	**–**	**–**	–	–
GH < 2.5 ng/ml + IGF-I < 1 ULN, *n* (%)	0 (0)	11 (39.3)	18 (66.7)	15 (83.3)	14 (87.5)	**–**	**0.04**	**–**	**–**	0.16	0.73
GH < 1 ng/ml + IGF-I < 1 ULN, *n* (%)	0 (0)	7 (25)	12 (44.4)	8 (44.4)	7 (43.7)	**–**	0.13	**–**	**–**	1	0.97
MTD, mm	17 ± 8.7	-	14.5 ± 6	12.6 ± 3.5	12.4 ± 3.7	**–**	–	** < 0.001**	**0.002**	0.19	0.36
Glucose profile											
FG, mg/dl	102.2 ± 15.2	117.1 ± 26.6	119.2 ± 36	118.9 ± 19	123.9 ± 20.6	** < 0.001**	0.78	**0.007**	**0.04**	0.65	0.32
FI, mU/l	8.3 ± 5.3	4.4 ± 2.6	4.1 ± 2.4	4.7 ± 2.3	4.7 ± 2.3	**0.004**	0.36	**0.003**	**0.003**	0.4	0.87
HbA1c, %	6 ± 0.7	6.1 ± 0.8	6.3 ± 0.9	6.1 ± 0.6	6.4 ± 0.7	**0.05**	0.31	**0.007**	0.97	0.29	**0.04**
NGT, *n* (%)	13 (46.4)	9 (32.1)	6 (22.2)	1 (5.5)	1 (6.2)	0.27	0.41	0.06	**0.003**	0.13	0.93
IFG, *n* (%)	7 (25)	5 (17.8)	6 (22.2)	2 (11.1)	2 (12.5)	0.56	0.68	0.80	0.25	0.34	-
DM, *n* (%)	8 (28.6)	14 (50)	15 (55.6)	15 (83.3)	13 (81.2)	0.10	0.68	0.04	**0.003**	**0.05**	0.87
Glucose Impairment, *n* (%)	15 (53.6)	19 (67.8)	21 (77.8)	17 (94.4)	15 (93.7)	0.27	0.41	0.06	**0.003**	0.13	0.93
HOMA-IR	2 ± 1.3	1.23 ± 0.9	1.2 ± 0.7	1.4 ± 0.7	1.45 ± 0.7	**0.004**	0.59	0.02	0.07	0.51	0.65
HOMA-β	99.7 ± 67.1	47.4 ± 46.9	36.4 ± 28.4	31.8 ± 17.7	31.2 ± 17.7	0.13	0.26	**0.004**	**0.004**	0.88	0.34
ISI_0_	16.6 ± 7.7	30.1 ± 22.2	43.7 ± 69.6	22.3 ± 10.9	21.1 ± 10.9	**0.006**	0.31	**0.006**	0.10	0.24	0.60
Lipid profile											
Total cholesterol, mg/dl	178.1 ± 39.6	182.6 ± 35.2	178.2 ± 31.1	176.2 ± 39.2	176.9 ± 35	0.20	0.46	0.20	0.43	0.59	0.44
HDL, mg/dl	51.2 ± 10.1	56.4 ± 9.8	50.9 ± 10.8	52.9 ± 9.7	58.2 ± 9.2	0.03	0.49	0.19	0.13	0.65	0.33
LDL, mg/dl	108 ± 34.7	109.7 ± 28.9	109.8 ± 27.8	105.4 ± 34.5	98.6 ± 6	0.54	0.12	0.38	0.50	0.27	0.47
Triglycerides, mg/dl	100.2 ± 50	91.4 ± 34.3	89.7 ± 33.8	94.2 ± 32.7	99.6 ± 32.7	0.55	0.98	**0.05**	0.62	0.91	0.53
Hypercholesterolemia, n (%)	13 (46.4)	12 (42.8)	8 (29.6)	11 (61.1)	9 (56.2)	0.79	0.31	0.20	0.33	0.04	0.77
Hypertriglyceridemia, n (%)	3 (10.7)	2 (7.1)	1 (3.7)	2 (11.1)	54 (25)	0.64	0.57	0.32	0.97	0.33	0.29

*MTD *maximal tumor diameter, *FG *fasting glucose, *FI *fasting insulin, *HbA1c *glycated hemoglobin, *NGT *normal glucose tolerance, *IFG *impaired fasting glucose, *DM *diabetes mellitus;

*Evaluation on 23 patients, 5 patients administered with pegvisomant were excluded

Based on FG, the diagnosis of impaired fasting glucose (IFG) and diabetes mellitus (DM) was performed according to WHO guidelines [14]. Insulin resistance was assessed using HOMA index in line with Matthews and coworkers, by calculating HOMA-IR = [insulin (mU/l) × FG (mmol/l)]/22.5 as surrogate index of insulin resistance, and HOMA-β = [20 × insulin (mU/l)/FG (mmol/l) –3.5] as surrogate index of insulin secretion, whereas ISI_0_ = 10,000/insulin (mU/ml) × FG (mg/dl) was used to assess baseline insulin sensitivity [15, 16].

Hypercholesterolemia and hypertriglyceridemia were diagnosed according to international guidelines [17, 18].

### Treatment protocol

According to the standard protocol of the center, prior to start of PAS treatment, first-line SRLs were administered at the initial dose of 20 mg/28 days for octreotide LAR or 120 mg/28 days for lanreotide [19]. To normalize GH and IGF-I levels, the SRLs dose was progressively increased, with dose adjustment being carried out every 3–6 months based on serum GH and IGF-I levels. Due to SRLs therapy resistance, seven patients (25%) were administered combined treatments (Table 1). Despite that, at baseline, 20 patients (71.4%) presented uncontrolled disease (IGF-I > 1.3 ULN) and 6 (21.4%) exhibited partial disease control (i.e., IGF-I 1–1.3 × ULN). To achieve a full disease control (i.e., IGF-I < 1 x ULN), these patients were administered with PAS, whereas two patients (7.1%) with normal IGF-I levels were switched to PAS due to side effects of the previous treatments. To achieve a better disease control, PAS was started at the dose of 20 mg/28 days in 2 (7.1%), 40 mg/28 days in 23 (82.1%), and 60 mg/28 days in 3 patients (10.7%), according to GH and IGF-I levels. PAS dose adjustment was subsequently carried out every 3–6 months based on serum GH and IGF-I levels.

### Assays

All biochemical and hormonal parameters were measured by standard methods, according to international standards. IGF-I is expressed in relation to the ULN to reduce the variability due to gender, age, and different assays.

### Statistical analysis

Data were analyzed using SPSS Software for Windows, version 28 (SPSS, Inc., Cary, N.C., USA). Data are reported as mean ± standard deviation (SD), unless otherwise specified.

The normal distribution of study variables was assessed with the Kolmogorov–Smirnov test. The comparison between the numerical data before and after treatment was made by a non-parametric Wilcoxon test for continuous variables. For those patients with available data at each time point of the study up to the LFU > 36 months, the comparison between the numerical data before and after treatment was made by a non-parametric Friedman test corrected by Dunn’s test, when necessary. The comparison between the numerical data between two different groups of patients was made by non-parametric *U* Mann–Whitney test. The comparison between prevalence was performed by Chi-squared test corrected by Fisher exact test when necessary. The correlation study was done by calculating Spearman’s correlation coefficients. Linear regression analysis was performed to investigate the best predictors of FG levels after 6 months of PAS therapy. Significance was set at 5%.

## Results

Complete biochemical, hormonal, and radiological parameters at each time point are detailed in Table 2.

The age at diagnosis of acromegaly was 40.5 ± 11.6 years and the disease duration before PAS treatment was 182.9 ± 82.7 months. At diagnosis, all but 2 patients harbored a pituitary macroadenoma (92.9%); 20 patients (74.1%) underwent neurosurgery as first-line treatment, whereas 8 patients (25.9%) were treated only with medical therapy. One patient (3.6%) undergone neurosurgery three times, and two patients (7.1%) received pituitary radiation therapy, specifically *Gamma-knife* radiosurgery, two and five years before PAS treatment, respectively.

### Baseline

GH and IGF-I levels were 4.1 ± 3.3 ng/ml and 458.4 ± 184.8 ng/ml (1.8 ± 0.6 × ULN), respectively. Concerning pituitary tumor, MTD was 17 ± 8.7 mm in the whole cohort and one patient (3.6%) had an empty sella. Glucose profile impairment was present in 15 patients (53.6%), particularly IFG was found in 7 (25%), whereas 8 (28.6%) had overt DM. Glucose-lowering treatments are detailed in Table 3. Patient’s age directly correlated with FG at baseline (r = 0.376, p = 0.05). Considering disease duration before PAS, FG (p = 0.01) and HbA_1c_ (p = 0.004) levels were significantly higher in patients with disease duration above as compared to those below the median (84 months).

**Table 3:** Glucose profile and glucose-lowering treatment of the entire cohort throughout the entire study. Patients are divided according to glycemic status at baseline

Patient n °	Age at pasireotide start, years	Baseline				6 months				12 months				36 months			
		Glycemic status	FG mg/dl	HbA1c %	Glucose-lowering treatment	Glycemic status	FG mg/dl	HbA1c %	Glucose-lowering treatment	Glycemic status	FG mg/dl	HbA1c %	Glucose-lowering treatment	Glycemic status	FG mg/dl	HbA1c %	Glucose-lowering treatment
**NGT at baseline**																	
**1**	37	NGT	94	5.3	NO	NGT	82	5.2	NO	NGT	91	5.3	NO	IFG	107	5.3	NO
**3**	43	NGT	87	5.6	NO	IFG	107	6	NO	IFG	125	6.3	NO	DM	128	6.4	Met 1000 mg/die
**12**	45	NGT	75	5.4	NO	IFG	114	5.3	NO	IFG	104	5.6	NO	DM	101	5.2	Met 1000 mg/die
**15**	31	NGT	98	5.6	NO	NGT	91	5.7	NO	NGT	94	5.6	NO	DM	108	5.8	Met 500 mg/die
**16**	56	NGT	88	6.1	NO	DM	132	5.9	NO	DM	127	6.2	Met 1000 mg/die	DM	129	5.9	Met 2000 mg/die
**18**	45	NGT	87	5.7	NO	IFG	79	5.4	Met 750 mg/die	NGT	79	5.4	NO	IFG	102	5.2	–
**20**	43	NGT	96	5.4	NO	IFG	102	5.5	NO	NGT	78	5.6	NO	–	–	–	–
**22**	55	NGT	88	5.2	NO	NGT	87	5.3	NO	IFG	100	–	NO	–	–	–	–
**23**	32	NGT	97	5.6	NO	NGT	97	5.9	NO	IFG	107	–	NO	–	–	–	–
**24**	39	NGT	91	5.3	NO	NGT	88	5.3	NO	NGT	81	5	NO	–	–	–	–
**26**	62	NGT	97	6.2	NO	IFG	114	–	NO	DM	121	7.2	NO	–	–	–	–
**27**	45	NGT	81	5.3	NO	NGT	98	5.5	NO	–	–	–	–	–	–	–	–
**28**	38	NGT	88	5.5	NO	NGT	75	5.3	NO	NGT	85	5.3	NO	–	–	–	–
**IFG at baseline**																	
**4**	28	IFG	104	5.4	NO	NGT	99	5.8	NO	NGT	87	5.8	NO	NGT	91	5.8	NO
**6**	52	IFG	108	6.4	NO	DM	140	6.5	Lira 0.6 mg/die	DM	120	6.5	Lira 0.6 mg/die	DM	121	6.3	Lira 1.2 mg/die
**7**	28	IFG	101	6	NO	DM	132	6.5	Met 1350 mg/die	DM	118	7	Met 1700 mg/die	DM	99	5.8	Met 2000 mg/die + Dula 0.75 mg/wk
**13**	43	IFG	102	5.8	NO	DM	121	6.3	Met 750 mg/die	DM	124	5.9	Met 1000 mg/die	DM	120	5.7	Met 1000 mg/die
**19**	37	IFG	100	5.7	NO	IFG	115	6.4	NO	IFG	115	6.4	NO	–	–	–	–
**17**	70	IFG	101	5.6	NO	DM	143	6.7	NO	DM	158	6.3	Met 2500 mg/die	DM	140	6.6	Met 2000 mg/die + Sita 100 mg/die
**25**	73	IFG	108	6.3	NO	DM	122	6.6	NO	DM	114	6.5	Met 2000 mg/die	–	–	–	–
**DM at baseline**																	
**2**	59	DM	131	7.1	Met 2000 mg/die + Sita 100 mg/die	DM	162	7.1	Met 2000 mg/die + Sita 100 mg/die	DM	131	7.2	Met 2000 mg/die + Lira 1.2 mg/die	DM	130	7.2	Met 2500 mg/die + Lira 1.8 mg/die
**5**	51	DM	139	7.7	Lira 1.2 mg/die	DM	146	7.7	Vilda100 mg/die	DM	126	7.6	Met 2000 mg/die + Vilda 100 mg/die	DM	118	6.5	Met 2000 mg/die + Vilda 100 mg/die
**8**	53	DM	118	5.8	Lira 1.2 mg/die	DM	120	5.7	Lira 1.2 mg/die	DM	120	5.8	Met 1000 mg/die + Sita 100 mg	DM	117	6	Met 1000 mg/die + Sita 100 mg/die
**9**	47	DM	106	6	Met 2000 mg/die + Sita 100 mg/die	DM	122	6.5	Met 2000 mg/die + Sita 100 mg/die	DM	125	6.4	Met2000mg/die + Sita 100	DM	140	6.9	Met 2500 mg/die + Lira 1.8 mg/die
**10**	67	DM	130	7.3	Met 2000 mg/die + Lira 1.8 mg/die	DM	163	7.3	Met 2000 mg/die + Lira 1.8 mg/die	DM	253	8.5	Met 2000 mg/die + Lira 1.8 mg/die	DM	95	6.5	Met 2000 mg/die + Lira 1.2 mg/die
**11**	59	DM	109	7.4	Met 1000 mg/die	DM	122	6.8	Met 2500 mg/die	DM	135	6.8	Met 2500 mg/die	DM	166	7.4	Met 2500 mg/die + Acarb 150 mg/die
**14**	50	DM	115	6.6	NO	DM	115	6.6	Met 1350 mg/die	DM	115	6.7	Met 1350 mg/die	DM	128	6.5	Met 1700 mg/die
**21**	51	DM	124	5.9	Met 1000 mg/die	DM	186	8	Met 2500 mg/die	DM	146	7.6	Met 2500 mg/die	–	–	–	–

Met = metformin, Sita = sitagliptin, Lira = liraglutide, Vilda = vildagliptin, Dula = dulaglutide, Acarb = acarbose

Concerning lipid metabolism, 13 patients (46.4%) had isolated hypercholesterolemia and 3 (10.7%) hypertriglyceridemia.

### Evaluation after 6 months

**Fig. 2:**
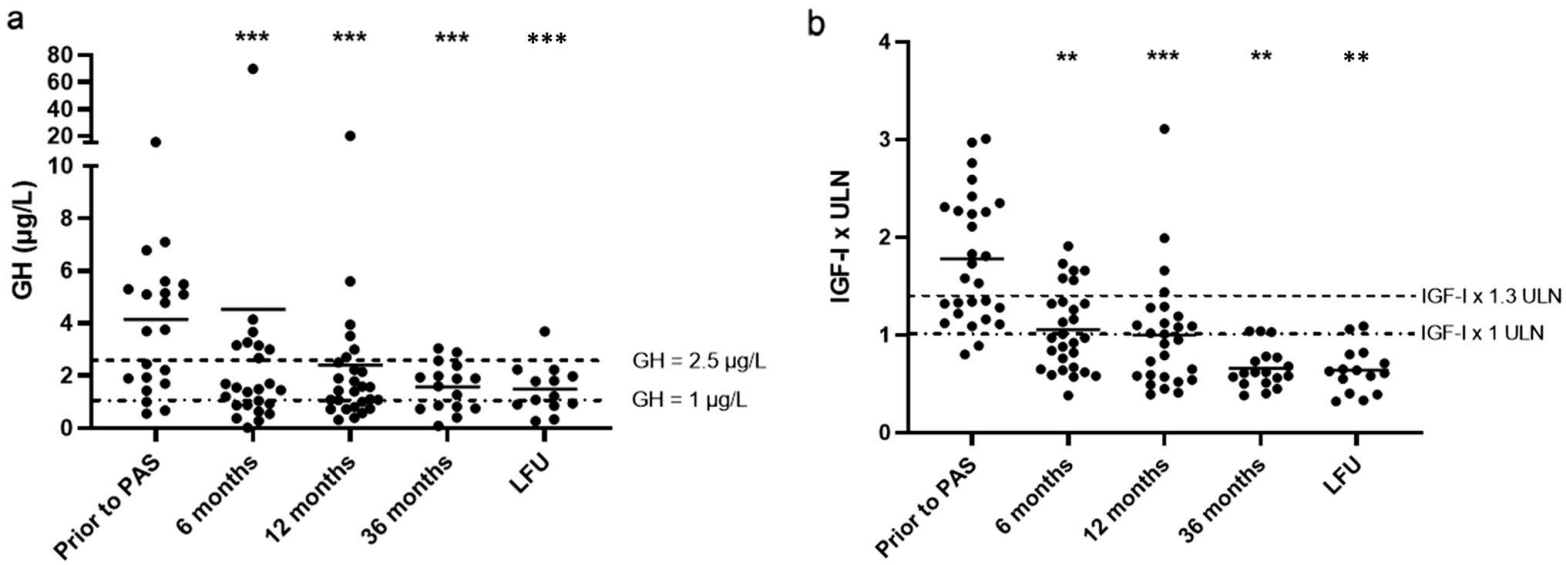
Changes in GH and IGF-I levels throughout the study. * are provided when a significant difference compared to baseline was found

Serum IGF-I levels were fully normalized in 16 patients (57.1%, Fig. 2b)*.* One patient required a decrease in PAS dose due to an excessive IGF-I reduction.

**Fig. 3:**
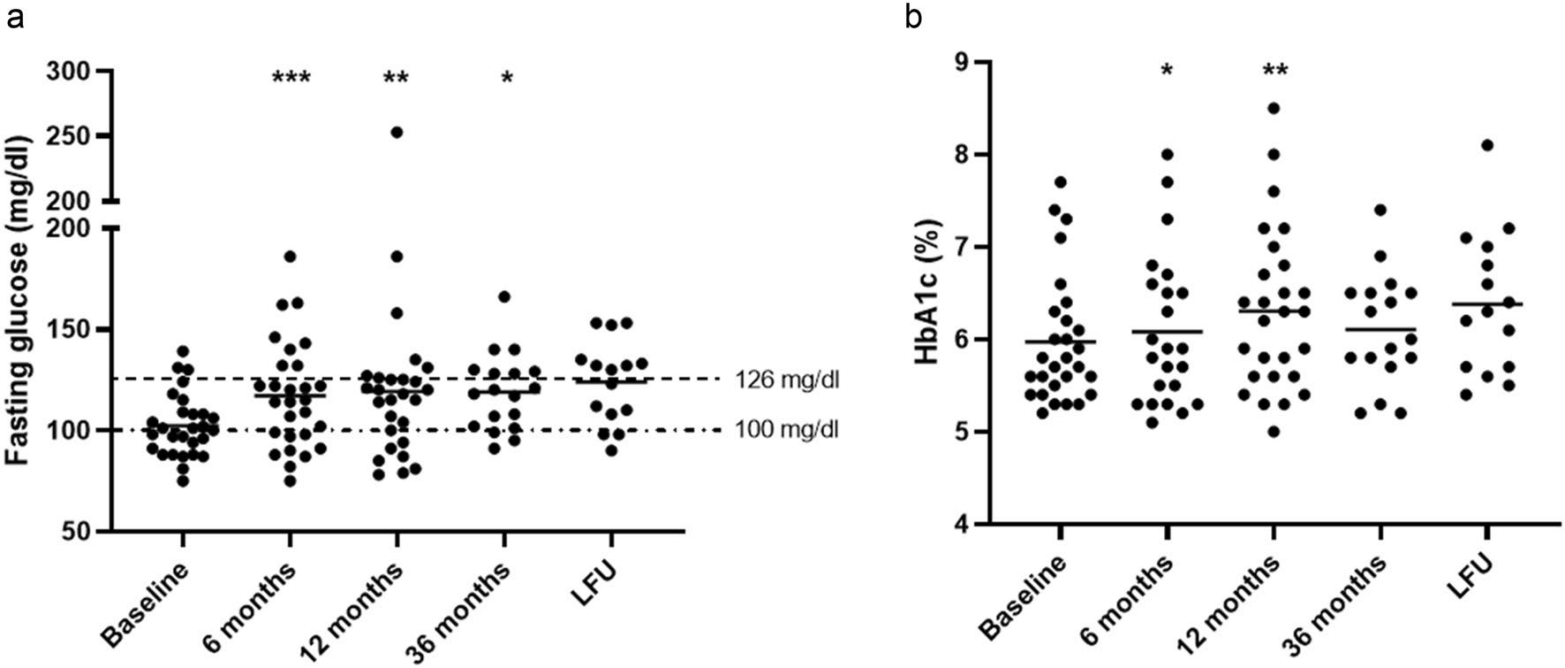
Changes in fasting glucose and HbA1c throughout the study. * are provided when a significant difference compared to baseline was found

**Fig. 4:**
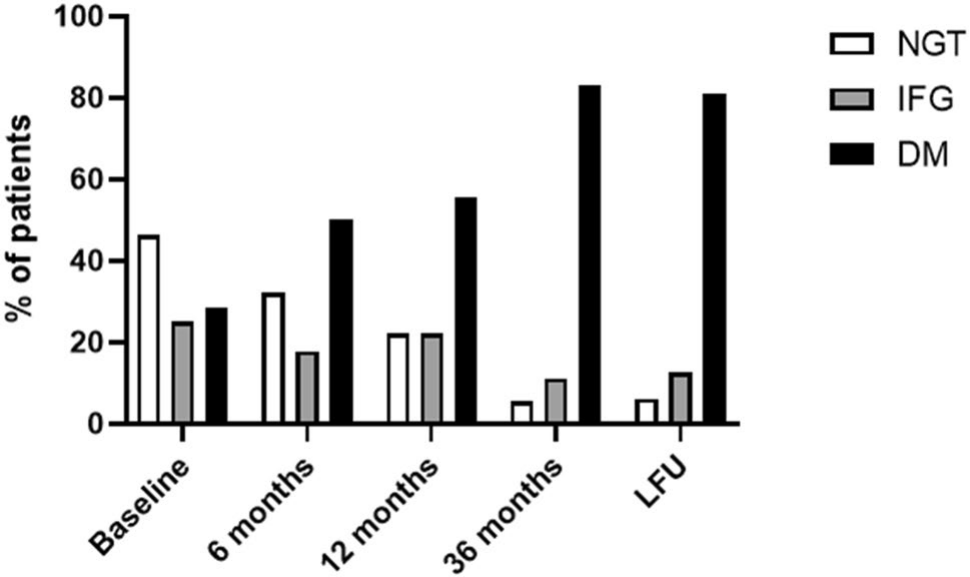
Prevalence of normal glucose tolerance (NGT), impaired fasting glucose (IFG), and diabetes mellitus (DM) throughout the study

FG significantly increased compared to baseline (117.1 ± 26.6 mg/dl *vs* 102.2 ± 15.2, *p* < 0.001, Fig. 3a), with a concomitant significant increase in HbA_1c_ levels (6.1 ± 0.8 *vs* 6 ± 0.7%, *p* = 0.05, Fig. 3b), but not in DM prevalence (50% *vs* 28.6%, *p* = 0.10, Fig. 4). At the regression analysis, FG at baseline was the best predictor of FG levels after 6 months of PAS therapy (β = 0.62, tau 4.04, *p* < 0.001). On the other hand, FI significantly decreased compared to baseline (*p* = 0.004), with consequent improvement in all insulin-derived indices (HOMA-IR, HOMA-β and ISI_0_, Table 2).

Among patients with normal glucose tolerance at baseline (NGT, no = 13), five (38.5%) developed IFG and one (7.7%) developed DM, whereas among patients with IFG at baseline (no = 7), five (71.4%) developed DM and in one (14.3%), the restoration of NGT was seen with the achievement of disease control (Table 3). Overall, 9 patients had NGT (32.1%), 5 showed IFG (17.8%), and 14 had DM (50%). Among patients who were not diabetic at baseline (no = 20), new-onset DM was found in six (30%). Glucose-lowering treatments required for DM management are detailed in Table 3.

Regarding lipid profile, a significant increase in HDL levels occurred (*p* = 0.03), without relevant impact of PAS on the other lipid fractions (Table 2).

### Evaluation after 12 months

IGF-I was fully normalized in 19 patients (70.4%, *p* < 0.001, Fig. 2b) without significant difference in prevalence of IGF-I control between patients previously undergone or not to neurosurgery (73.7% *vs* 55.5%, *p* = 0.34). Furthermore, per cent change (Δ%) of IGF-I during the first 12 months of PAS was inversely correlated to IGF-I levels at baseline (*r* = − 0.433, *p* = 0.02). Considering disease duration before PAS, Δ%IGF-I during the first year of PAS was lower in patients with disease duration above as compared to those below the median (mean − 31% *vs* − 41%, *p* = 0.06).

**Fig. 5:**
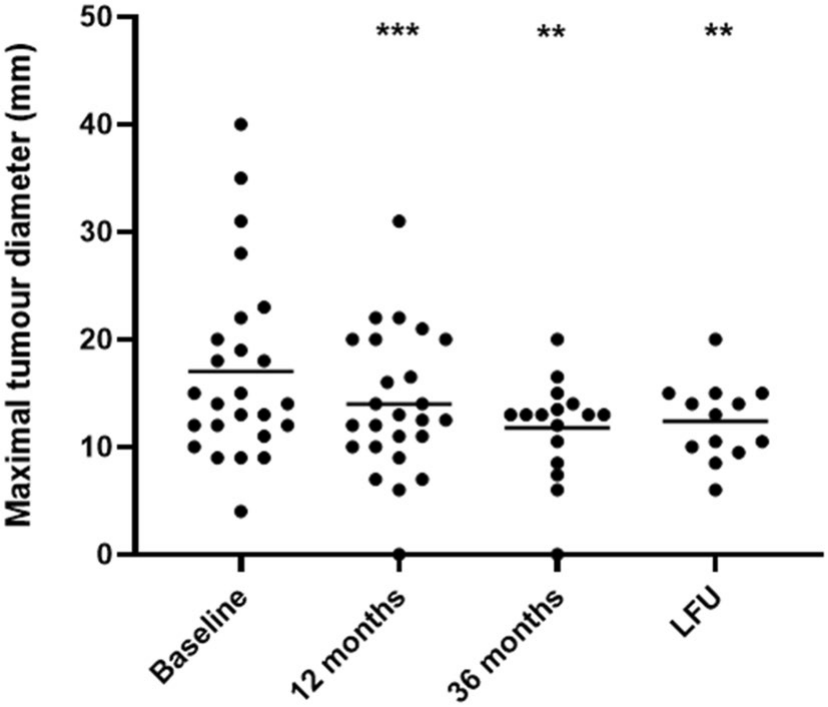
Changes in maximal tumor diameter throughout the study. * are provided when a significant difference compared to baseline was found

Apart from one patient with empty sella, MTD was significantly reduced in the entire cohort (*p* < 0.001, Fig. 5); a significant decrease was found in four patients (14.8%). Notably, Δ%MTD was similar between patients with IGF-I < 1 × ULN and patients still not fully controlled (− 13.7% vs − 16.2%), suggesting that PAS is able to induce tumor shrinkage regardless from the full biochemical disease control.

**Fig. 6:**
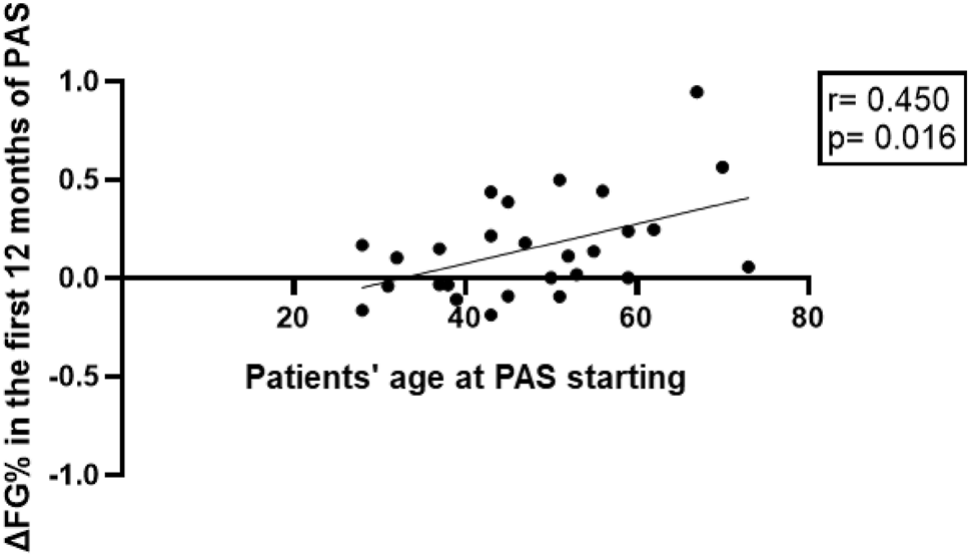
Per cent change (Δ%) in fasting glucose in the first 12 months of pasireotide (PAS) was directly correlated to patient’s age at pasireotide starting

DM prevalence was significantly increased compared to baseline (55.6% *vs* 28.6%, *p* = 0.04, Fig. 4), although with controlled HbA_1c_ levels (mean 6.3%, Fig. 3b). As a consequence, an increased number of glucose-lowering drugs was necessary compared to baseline (*p* = 0.07), details are shown in Table 3. FG (*p* = 0.02) and HbA_1c_ (*p* = 0.003) levels persisted significantly higher in patients with disease duration before PAS above as compared to those below the median (84 months). Furthermore, patient’s age at PAS starting was directly correlated to Δ%FG during the first 12 months of PAS (*r* = 0.450, *p* = 0.01, Fig. 6). Indeed, seven patients (25%) exhibited a reduction in FG at 12-month evaluation. These patients were significantly younger both at acromegaly diagnosis (*p* = 0.01) and at baseline (*p* = 0.01) compared to patients who experienced a worsening of FG during the first 12 months of PAS.

Triglycerides were significantly reduced compared to the levels prior to PAS treatment (*p* = 0.05), without significant changes in the other lipid fractions.

### Evaluation after 36 months

Eighteen patients (64.3%) received PAS for 36 months (Fig. 1)*.* All these patients exhibited fully normalized IGF-I (mean IGF-I 0.7 × ULN, Fig. 2b), without requiring a further increase in PAS dose after the first 12 months of therapy. Interestingly, patients administered with PAS starting dose of 60 mg every 4 weeks exhibited significantly lower IGF-I levels (*p* = 0.05) as compared to the patients who started with PAS 20 or 40 mg, resulting PAS dose administered in the first 6 months inversely correlated to IGF-I levels at 36 months (*r* = − 0.524, *p* = 0.02). Thus, suggesting that the use of high PAS dose since the start of treatment might help in inducing a greater reduction in IGF-I levels after a long treatment duration.

Considering tumor size, MTD was further reduced and a significantly higher number of patients achieved a decrease in MTD > 25% compared to 12-month evaluation (41.2% *vs* 14.8%, *p* = 0.05). Furthermore, disease duration before PAS was inversely correlated to Δ%MTD from baseline (*r* = − 0.492, *p* = 0.06), suggesting that the prompt switch to PAS in patients with acromegaly resistant to first-generation SRLs might promote a greater tumor shrinkage. Therefore, besides the early achievement of biochemical control, PAS seems also to exert a prolonged anti-proliferative effect.

All but three patients were diabetic (83.3%), resulting in a further significant increase in DM prevalence as compared to 12-month evaluation (*p* = 0.05, Fig. 4). However, patients’ glycemic control, expressed by HbA_1c_, remained stable compared to levels prior to PAS (*p* = 0.97, Fig. 3b). Particularly, Δ%FG was slightly higher in patients previously treated with SRLs and PEG combined treatment as compared to those receiving SRLs monotherapy (*p* = 0.08). Overall, 15 patients had DM (83.3%), two showed IFG (11.1%) and only one (5.5%) had NGT (Fig. 4***)***. The patient with NGT (n°14, Table 3) was the youngest of the entire cohort, who showed IFG before PAS treatment and normal FG levels with the achievement of disease control after 6 months of PAS. Noteworthy, among patients with NGT at baseline who were treated with PAS for 36 months (no = 6), two (33.3%) developed IFG and four (66.7%) developed an overt DM. Consequently, a significant increase in the number of glucose-lowering drugs was observed after 36 months of treatment compared to baseline (*p* = 0.002), details are shown in Table 3.

### Last follow-up evaluation

Among patients who entered this study, 16 (57.1%) were treated with PAS for a period longer than 36 months (mean 61 months, range 42–90). All these patients exhibited optimal disease control, being mean IGF-I as high as 0.6 × ULN and pituitary tumor size stable as compared to the 36-month evaluation (Figs. 2b, 5). Moreover, all metabolic parameters were stable as compared to the 36-month evaluation and no further change in antidiabetic medications was required. Overall, NGT, IFG, and DM were recorded in 6.2%, 12.5%, and 81.2% of patients, respectively (Fig. 4).

## Discussion

The current retrospective study of data prospectively collected has investigated the impact of long-term PAS therapy on disease control, pituitary tumor size, gluco-insulinemic and lipid profile in PAS naïve acromegaly patients in a real-life setting.

Previous studies investigating prolonged therapy with PAS have reported the great efficacy of PAS in the early achievement of GH and IGF-I normalization, together with disease control maintenance over time. Indeed, *PAOLA* extension study has reported that 30% and 50% of patients treated with PAS 40 mg and 60 mg, respectively, were still controlled at the end of the extension phase (268 weeks) [10]. Scant real-life data about long-term PAS treatment are available to date, one multi-centre retrospective study reported disease control in 79% of patients after a mean treatment duration of 50 months [11]. Notably, this study included 19 patients, among whom three were already treated with PAS as part of the previous randomized phase III trial whereas 16 were first administered with PAS. Similarly, another recent study included 27 patients receiving long-term PAS (up to 11 years) and reported the achievement of disease control in 54% after a median duration of 58 months [20]. Available studies regarding long-term PAS therapy are summarized in Table 4.

**Table 4:** Overview of currently available studies about long-term pasireotide therapy

First author, year	Study type	Follow-up duration (months)	IGF-I < 1 × ULN (%)	Decrease in pituitary tumor size > 25% (%)	New onset diabetes (%)	Pasireotide withdrawal due to hyperglycemia (%)
*Colao *et al*., 2020*	Clinical trial, extension of a prospective, randomized phase III study	67 months	30% of patients treated with 40 mg/monthly 50% of patients treated with 60 mg/monthly	NA	31.7% of patients treated with 40 mg/monthly 40.3% of patients treated with 60 mg/monthly	NA
*Akirov *et al*., 2021*	Retrospective study evaluating patients naive to pasireotide or previously enrolled in randomized phase III trials	Up to 135 months	79%	NA	31.6%	15.8%
*Gadelha *et al*., 2023*	Retrospective study of data prospectively collected of patients enrolled in clinical studies with pasireotide	Up to 137 months	54%	62%*	30%	12%
*Pirchio *et al*., 2024 current study*	Retrospective study of data prospectively collected in real-life setting	Up to 90 months	93.7%	41.2%	52.6%	0%

^*^data not available for all the patients

Current results confirmed PAS to be very effective in the achievement of disease control, with IGF-I being fully normalized in 70.4% of patients after 12 months of treatment and in the entire cohort after 36 months of treatment. Therefore, disease control prevalence resulted to be higher than that reported in clinical trials [5, 6] but similar to the real-life multi-centre retrospective study [11]. Beyond the great efficacy of PAS *per se*, this promising result could be also ascribed to the careful and targeted selection of patients, the prompt dose adjustment, the early discontinuation and the switch to other therapies in the four (14.3%) non-responder patients. Moreover, IGF-I levels after 36 months of treatment were demonstrated to be inversely correlated to the PAS dose administered in the first 6 months of therapy, suggesting that starting PAS therapy with a higher dose could be useful to rapidly lower IGF-I levels with long-term effects, leading to a better disease control.

According to the recent acromegaly consensus statement [2], PAS is approved as third-line treatment in patients resistant to first-generation SRLs, mainly in those with clinically relevant residual tumor and/or clinical concern of tumor growth [1], as PAS can influence tumor cell growth. The high rate of tumor shrinkage observed after the use of PAS has been demonstrated in a previous study [7], in which a greater reduction in tumor volume was achieved in patients treated with PAS as compared to patients treated with octreotide LAR. Consistently, in the current study, MTD was significantly reduced after 12 months of PAS treatment in the entire cohort, being a significant decrease in MTD achieved in about 14.8% of patients in the first 12 months of treatment. Furthermore, tumor shrinkage was found to be sustained and prolonged, being the prevalence of a significant decrease in MTD as high as 41.2% after 36 months of PAS. The reasons for this phenomenon need to be further elucidated; however, a potential direct impact of PAS treatment on tumor consistency can be hypothesized, as previously demonstrated for first-generation SRLs. The use of preoperative SRLs has been indeed reported to affect tumor consistency in GH-secreting macroadenomas, although with discordant results [21], and to increase the success rate of neurosurgery [21–23]. In the current study, a relevant tumor shrinkage induced by PAS was observed in patients not undergone pituitary surgery. Furthermore, two patients experienced a successful neurosurgery, after achieving a significant reduction in pituitary adenoma size with PAS. These experiences may encourage the use of PAS before neurosurgery in patients with large tumor masses not candidated for gross-total removal, as for octreotide LAR. Future research will help in elucidating the potential preoperative role of PAS in acromegaly.

Moreover, the current study first demonstrated that pituitary tumor shrinkage was prolonged and sustained, even after the achievement of disease control and without requiring further increase in PAS dose. These findings suggest that, besides the early achievement of disease control and the consequent improvement of acromegaly symptoms and quality of life, PAS could exert a direct and prolonged anti-proliferative action. Hence, the patient can benefit from both the rapid normalization of IGF-I levels and the long-term anti-proliferative effect of PAS therapy. Furthermore, the per cent change in MTD found in this study was demonstrated to be inversely correlated to disease duration before PAS, suggesting that a prompt use of PAS at the higher doses in patients resistant to first-generation SRLs could allow the achievement of greater tumor shrinkage, with no relevant concerns on the potential hyperglycemic effects, since no direct relation between PAS dose and the grade of hyperglycemia has been documented in the present or in previous studies [24].

In fact, PAS-induced hyperglycemia still represents the main concern, being the most common adverse event during this treatment. The occurrence of hyperglycemia has been reported in about 60% of patients in *C2305* [5] and *C2402* [6] clinical trial studies. Particularly, grade 3 and 4 hyperglycemia (i.e., clinically relevant) was found to occur in 3.4% of PAS-treated as compared to 0.6% of those octreotide LAR-treated for patients naïve to medical therapy [5], and in 9.6% of PAS-treated as compared to 0% of first-generation SRLs treated for patients resistant to first-generation SRLs [6]. Similarly, in real-life settings, the increase in the prevalence of glucose impairment has also been observed after the first months of PAS treatment, requiring a significant intensification of glucose-lowering therapies [9]. In line with this evidence, the results of the current study showed a significant increase in FG within the first 12 months of PAS therapy, with a concomitant increase in IFG and DM prevalence, whereas HbA_1c_ levels remained quite stable throughout the study period, suggesting that PAS exerts a negative impact mainly on morning FG. Although not statistically significant, prevalence of DM increased from 28.6% at baseline to 50% at 6 months evaluation, confirming previous data about the early onset of hyperglycemia and the consequent need for a strict glucose monitoring during the first months of PAS treatment. These findings are in line with previous data, reporting DM prevalence to increase from 14.3% to 47.6% after 6 months of PAS treatment [25]. Notably, glucose impairment, which has been demonstrated to be the main predictive factor of glycemic worsening, was already present at baseline in about 50% of the patients enrolled in the current study. Despite the high prevalence of pre-existing glucose metabolism impairment, optimal glycemic control was reported during the study in the whole cohort thanks to the proper adaptation of glucose-lowering drugs throughout the years. Notably, all the patients were treated with oral glucose-lowering drugs or with glucagon-like peptide 1 (GLP-1) agonists, without requiring insulin administration. Consequently, no patient of the current series required PAS withdrawal due to hyperglycemia-related adverse events. Hence, prompt and tailored adjustment of the common glucose-lowering drugs allowed the administration of PAS in all patients who can benefit from this treatment, regardless of pre-existing alterations in glucose metabolism.

Based on previous clinical trial findings, several risk factors for glycemic worsening have been identified. Indeed, old age and the presence of pre-existing impaired glucose tolerance, dyslipidemia, and hypertension appeared to increase the likelihood of developing hyperglycemia during PAS treatment [24]. In line with previous results, age and FG levels at PAS therapy initiation have been confirmed to be predictive factors of DM development in the current study. Therefore, older patients with concomitant risk factors should deserve a careful and strict monitoring of FG during the first months of therapy to early detect the onset of DM and to start promptly the administration of glucose-lowering drugs. The beneficial role of the young age on the likelihood of developing hyperglycemia during PAS treatment is supported by the case of the youngest patient of the current series, who showed IFG before PAS and achieved NGT after IGF-I normalization while on PAS therapy and by the finding of a significant correlation between patient’s age at PAS starting and Δ%FG during the first 12 months of PAS.

The pathogenesis of PAS-induced hyperglycemia is mainly ascribable to its peculiar receptor binding profile. Indeed, SSTRs are expressed in pancreatic cells and are involved in the regulation of insulin and glucagon secretion. Particularly, insulin secretion is mediated by both SSTR2 and SSTR5 [26], whereas glucagon secretion is mediated mainly by SSTR2 [21]. Since the affinity of PAS is higher for SSTR5 than for SSTR2, the suppression of insulin secretion is greater than that of glucagon, resulting in increased FG levels [27]. In healthy volunteers, PAS has been shown to inhibit not only insulin secretion, but also to reduce incretin response due to GLP-1 and glucose-dependent insulinotropic peptide (GIP) secretion decrease, with negligible inhibition of glucagon secretion and no impact on insulin sensitivity [28, 29]. Noteworthy, clinical trials [5, 6] investigating the efficacy and safety of PAS in either naïve or SRLs-resistant patients did not specifically report the outcome of PAS treatment on insulin and incretins secretion. To date, only one study investigated the impact of PAS on β-cell function in patients with acromegaly [30], demonstrating that the worsening of glucose homeostasis is mainly ascribable to a decrease in insulin secretion, without a substantial impact on insulin sensitivity. The results of the current studies are in line with previous preclinical studies [27, 31] and in part with the real-life study [30] as a significant decrease in FI levels has been reported throughout the study together with a decrease in the insulin-secretion index and an improvement in insulin resistance and sensitivity indices. A clear correlation between FI and changes in FG levels has been not likely observed in this study due to the small cohort.

In the current study, previous therapies for acromegaly have been demonstrated to influence the extent of the changes in glucose profile due to PAS. The use of SRLs in acromegaly has been reported to exert a non-significant beneficial effect on FG [32], mainly in patients achieving disease control [33]. In turn, PEG has been extensively reported to improve glucose and insulin levels, both directly and indirectly through IGF-I normalization [34–36]. Probably in the current study, the patients previously administered with SRLs combined with PEG lost the metabolic benefit of PEG on glucose metabolism after initiation of PAS, experiencing a greater increase in FG.

The management of PAS-associated hyperglycemia in acromegaly is still challenging. Previous studies concluded that PAS-induced DM should be managed by the initiation of medical therapy with metformin and staged treatment intensification with a DPP-4 inhibitor, with a switch to a GLP-1 receptor agonist and initiation of insulin, as required, to achieve and maintain glycemic control [37, 38]. Patients with new-onset DM following PAS therapy in the current study have been managed accordingly, starting with metformin and adding a DPP-4 inhibitor or GLP-1 receptor agonist if required. Notably, all patients were treated only with oral glucose-lowering drugs or GLP-1 agonists, without requiring insulin treatment. In these patients, oral glucose-lowering drugs allowed to maintain optimal control of DM, being the mean HbA_1c_ under 7% throughout the study. Furthermore, since FG and HbA_1c_ levels resulted to be significantly higher during the study in patients with disease duration before PAS above the median, the prompt starting of PAS in patients resistant to first-generation SRLs could also allow to a better glycemic control.

The impact of PAS on lipid profile in patients with acromegaly is yet to be fully elucidated. PAS has been shown to determine a great reduction in triglycerides but a modest decrease in total cholesterol levels in healthy volunteers in phase I study [39], and similar results have been reported in patients with Cushing’s disease after 12 months of treatment with subcutaneous PAS [40]. To the authors’ knowledge, no data are currently available regarding the effects on the lipid profile of PAS treatment in patients with acromegaly; therefore, this is the first study investigating this aspect. Overall, a significant reduction in triglycerides and increase in HDL levels during the first 12 months of PAS have been reported in the current study, together with a slight but not significant decrease in hypercholesterolemia and hypertriglyceridemia prevalence. Further studies are required to better clarify the effect of PAS on lipid profile in acromegaly.

The main limitations of the current study are the small number of patients considered and the different length of follow-up due to the starting of PAS in different periods, according to the real-life clinical practice.

Although the small number of patients due to the rarity of the disease and the limited number of patients requiring second- or third-line therapy, this is the first monocentric real-life study investigating the impact of long-term PAS therapy on disease control, pituitary tumor size, gluco-insulinemic and lipid profile in PAS naïve acromegalic patients in a real-life setting. Further studies on larger cohorts are required to confirm and deepen the results of the current research.

## Conclusion

The current is the first study evaluating patients with acromegaly treated with PAS in a real-life setting, reporting that PAS therapy is effective and safe in the long term. After the first 12 months of PAS, 70.4% of patients achieved normal IGF-I levels, which was maintained after 36 months of treatment in all these responder patients. Furthermore, PAS determined a significant tumor shrinkage in several patients in the first 12 months of treatment, which continued during the following months, resulting in the achievement of a significant decrease in maximal tumor diameter in 41.2% of patients after 36 months of PAS. Hence, acromegaly patient can benefit from both the rapid normalization of IGF-I levels and the long-term anti-proliferative effect of PAS therapy. Despite the likelihood of FG worsening and consequent onset of glucose impairment with PAS, optimal glycemic control can be obtained using oral glucose-lowering drugs or GLP-1 agonists in PAS-treated patients. Consequently, PAS could be safely administered to all acromegaly patients who could benefit from it, regardless from glycemic status before PAS. As worsening of FG levels has been demonstrated to occur mainly in the first 12 months of therapy, strict glucose monitoring should be adopted during this period. PAS could be administered also to patients already diabetic, who require careful monitoring and prompt adjustment of glucose-lowering drugs. Attention should be particularly paid to elderly patients with concomitant risk factors, in whom the worsening of FG levels to PAS starting might be more severe than in young patients. Since PAS starting dose exerted a beneficial impact on IGF-I levels in the long term without a dose-related hyperglycemic effect, choosing to start PAS therapy with a high dose could be an useful and safe approach to improve biochemical control in acromegaly patients with resistance to first-generation SRLs. Further, since the extent of IGF-I decrease was demonstrated to be lower in patients with a longer disease duration, PAS should be promptly started in patients with acromegaly resistant to first-generation SRLs to achieve a better disease control.

## Data Availability

The datasets generated during and/or analyzed during the present study are not publicly available but are available from the corresponding author on reasonable request.
